# Effect of Different Concentrations of Silver Nanoparticles on the Quality of the Chemical Bond of Glass Ionomer Cement Dentine in Primary Teeth

**DOI:** 10.3389/fbioe.2022.816652

**Published:** 2022-03-07

**Authors:** Faisal Mohammed Abed, Sunil Babu Kotha, Haneen AlShukairi, Fatmah Nasser Almotawah, Rwan Abdulali Alabdulaly, Sreekanth Kumar Mallineni

**Affiliations:** ^1^ Ministry of Health Specialized Dental Center, King Fahd General Hospital, Madinah, Saudi Arabia; ^2^ Department of Preventive Dentistry, College of Dentistry, Riyadh Elm University (REU), Riyadh, Saudi Arabia; ^3^ Department of Pediatric and Preventive Dentistry, Sharad Pawar Dental College and Hospital, Datta Meghe Institute of Medical Sciences (Deemed to be University), Wardha, India; ^4^ Dental Intern, College of Dentistry, King Saud University, Riyadh, Saudi Arabia; ^5^ Department of Preventive Dental Sciences, College of Dentistry, Majmaah University, Al-Majmaah, Saudi Arabia; ^6^ Center for Transdisciplinary Research (CFTR), Saveetha Institute of Medical and Technical Sciences, Saveetha Dental College, Saveetha University, Chennai, India

**Keywords:** bong strength, silver nanoparticles, bonding, glass ionomer, primary teeth

## Abstract

**Background:** The nanotechnologies have been applied for dental restorative materials manufacturing such as glass ionomer cement, composites, tooth regeneration, and endodontic sealers. The study aimed to investigate the chemical bond of conventional glass ionomer cement and to evaluate the addition of different concentrations of silver nanoparticles (AgNPs) on the quality of the chemical bond of glass ionomer cement to primary dentin.

**Methods:** Silver nanoparticle (AgNP) powder was added in concentrations of 0.2, 0.4, and 0.6% to the conventional powder of GIC Fuji II. Then, the powder was added to the liquid and mixed with the recommended powder/liquid ratio of 3.6:1 g. The Fourier-transform infrared spectra (FTIR) of teeth with 0.2%, 0.4%, and 0.6% w/w of silver nanoparticles in GIC fills and the control tooth were obtained. The conventional glass ionomer was used as a control group. The control and the plain silver tooth were subjected to FTIR analysis using an ATR–FTIR spectrophotometer (ThermoFisher Scientific, Waltham, MA, United States) with zinc selenide (ZnSe) ATR crystal (attenuated total reflection) and OPUS v7.5 software. All spectra were recorded in the range of 500–3,500 cm^−1^ in the transmission mode with an ATR module.

**Results:** The AgNPs added at 0.2, 0.4, and 0.6% concentration to GIC provided some information in the context of bond interaction with the dentin. Various bond peaks were seen for calcium, carbonate, phosphate, and amide. In our study, only the amide and phosphate were generated. The amide peaks were almost similar to the control, 0.2%, 0.4%, and 0.6%, with the peaks in the range of 1250–1650 cm^−1^. There was a clear shift in the phosphate peak from the control, 0.2, and 0.4%, which was about 1050 cm^−1^, whereas for 0.6%, there was a clear shift from 1050 cm^−1^ to 880 cm^−1^.

**Conclusion:** GIC supplemented with AgNPs showed that a concentration above 0.4% of AgNPs altered the bond quality in dentin interaction. In conclusion, adding AgNPs at a minimal level improves the mechanical properties and maintains the same bond quality as GIC.

## Introduction

The glass ionomer cement (GIC) was first discovered by Wilson and Kent (1972). It has been widely used for restorations, liners and bases, pit and fissure sealants, luting materials, core buildups, and orthodontic bracket adhesives (Cibim et al., 2017). GIC’s shortcomings are little fracture toughness, little wear resistance, and formal dissolution on water sorption that might lead to the growth of secondary caries, bacteria, and in the end, failure of the restoration (Garcia-Contreras et al., 2015). Furthermore, GICs have good biocompatibility, a low thermal expansion coefficient, and fluoride-releasing properties (Garcia-Contreras et al., 2015).

The secondary caries was reported as being the primary reason for the failure of GICs because the fluoride release was not enough to inhibit bacterial growth (Xie et al., 2011). The primary cause for caries and cariopathogenic biofilm development can be adhesion to the tooth surface by specific oral bacteria (Garcia et al., 2016). It can occur after a minimally invasive technique that would leave caries-affected tissues behind, thus resulting in elevation of the probability of residual bacteria on the prepared teeth cavities (Doozandeh et al., 2015). Furthermore, bacteria might invade tooth restoration interfaces throughout service when microleakage occurs in that region (Jowkar et al., 2019). Accordingly, a restoration may be affected by secondary caries that results from the growth of bacterial colonies, notably *Streptococcus mutans*, beneath the restorations (Kasraei et al., 2014).

For minimizing secondary caries failure, additional filler was introduced to improve the antibacterial and mechanical proprieties of the GICs without any interference with their bond strength and fluoride-releasing properties (Xu and Burgess, 2003; Garcia-Contreras et al., 2015). Nanotechnology is the science of producing functional structures and materials that range from 0.1 to 100 nm utilizing different physical and chemical processes. The developments of nanocomposites was the first attempt in the restorative dentistry field to use nanoparticles (NPs). This attempt has enabled scientists to develop nanoparticle-enriched GICs (Mitra et al., 2003). The nanotechnologies were applied for dental restorative materials manufacturing such as glass ionomer cement (i.e., nano-ionomers), composites (i.e., nanocomposites), tooth regeneration, and endodontic sealers (Mitra et al., 2011). The vital contribution from nanodental materials can be considered to be identifying oral health-related disorders *via* enhanced management and diagnosis of dental problems *via* bionanomaterials (Maman et al., 2018). Silver can be used in elementary and ionized forms such as nanoparticles or silver zeolites (Monteiro et al., 2012; Padovani et al., 2015; Köroğlu et al., 2016; Crystal and Niederman, 2019). A silver alloy powder was formerly added to a restorative glass ionomer cement to make a metal reinforced GIC, which is more complex and more substantial. A silver powder was sintered to glass at high temperatures to obtain cermet cement. It has been claimed that such silver-sintered powder could improve abrasion resistance and durability (Simmons, 1983; McLean et al., 1994).

Silver nanoparticle incorporation into GIC powder could reduce biofilm formations that would not significantly affect the mechanical and physical properties. In one study, silver nanoparticles were not firmly bonded to the matrix. They did not significantly improve the mechanical properties, which could be due to their nanosize, which allowed dispersion between and around polymer chains (Köroğlu et al., 2016; El-Wassefy et al., 2018; Crystal and Niederman, 2019). Incorporating silver nanoparticles into glass ionomer cements significantly enhanced the material’s wear resistance. The main improvement after adding silver nanoparticles was abrasion resistance and radio-opacity to the glass ionomer cement (McKinney et al., 1988; Xie et al., 2000). Fourier-transform infrared spectroscopy (FTIR) qualitative analysis can provide wave modes of molecules, assessed *via* the sample optical absorptions bands, which can be thought of as the fingerprints of specific molecules that provide accurate data on chemical changes inside a material. The latter being evaluated has suggested potential changes in absorption bands and/or new bands (Yamakami et al., 2018). Thus, there is evidence from previous studies about the advantages of adding silver nanoparticles (AgNPs), which show increased mechanical and antibacterial properties. Still, there are no studies on the quality of the bond interaction of silver nanoparticles (AgNPs) with dentin. The study aimed to investigate the chemical bond of conventional glass ionomer, evaluate the addition of silver nanoparticles (AgNPs) to traditional glass ionomer cement (GIC), and assess the effect of different concentrations of silver nanoparticles (AgNPs) on the quality of the chemical bond of glass ionomer cement to primary dentin.

## Methodology

### Materials

GC Fuji II [powder 15 g: 95% by weight alumino-fluoro-silicate glass with 5% polyacrylic acid powder, liquid 8 g (6.4 ml): 50 percent distilled water, 40 percent polyacrylic acid, and 10 percent polybasic carboxylic acid (GC, Tokyo, Japan)] and silver nanoparticles (AgNPs) (<100 µm in size) from Sigma Aldrich (St. Louis, MO, United States, Lot # MKBN3581V).

### Ethical Approval

The proposal was registered with the research center of Riyadh Elm University (FPGRP/43835007/334), and ethical approval was obtained from the Institutional Review Board of the institution.

### Preparation of Samples

**FIGURE 1:**
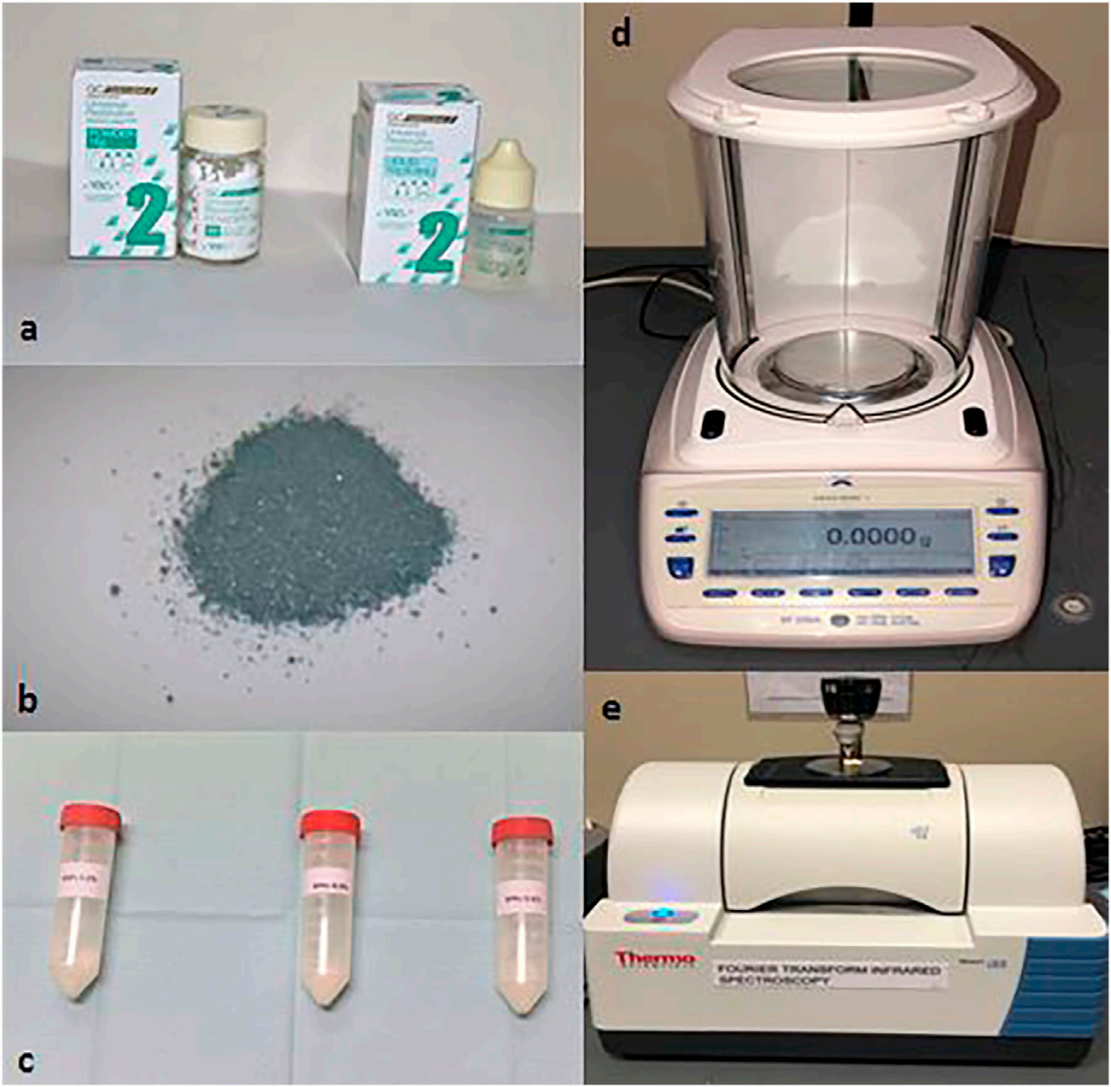
**(A)** GC Fuji II powder and liquid; **(B)** silver nanoparticle (AgNP) powder used for making samples; **(C)** mixed GIC powder and silver nanoparticle (AgNP) powder with different concentrations 0.2, 0.4, and 0.6%; **(D)** electronic weighing scale used for measuring the weight of the GIC powder and other substance(s); **(E)** Fourier-transform infrared spectroscopy (FTIR).

In this *in vitro* study, a conventional GIC (GC Fuji II, GC Corporation, Tokyo, Japan) (f) and silver nanoparticle powders (AgNPs) <100 nm particle size (Sigma-Aldrich, St. Louis, MO, United States) (Figure 1) were purchased. The SNP powder was weighed carefully using a weighing machine with an accuracy of ±0.0001 g Precisa (360A, Livingston, U.K) (Figure 1), and three concentrations were obtained: 0.2, 0.4, and 0.6% (w/w). The GIC specimens were divided into four groups for each test: GIC without silver nanoparticles (AgNPs) (*n* = 10), GIC with 0.2% silver nanoparticles (AgNPs) (*n* = 10), GIC with 0.4% silver nanoparticles (AgNPs) (*n* = 10), and GIC with 0.6% AgNPs (*n* = 10) (Figure 3). The materials were mixed with a powder/liquid P/L ratio of 2.6.1 g and were prepared following the manufacturer’s instructions.

### Preparation of Group(s)

The extracted teeth were collected from an operating room and dental clinic. The teeth were carefully examined to ensure the absence of debris. For 1 month, the teeth were stored in a 0.1% thymol solution with 0.9% isotonic sodium chloride (5°C) until the beginning of the experiment. We used a diamond separating disc (Edenta ISO No. 806.104.355.514.220, Switzerland; 15.000 rotations/min) at a slow-speed handpiece with continuous water cooling, perpendicular to the tooth’s long axis, and sectioned approximately 2.0 mm of the tissue along with the cusps without exposing the pulp (Porenczuk et al., 2016). The 40 teeth were categorized into four groups with an equal distribution that includes group 1 (apply GIC on dentin as the control group), group 2 (apply GIC with silver nanoparticles (AgNPs) (0.2%) on dentin), group 3 (apply GIC with silver nanoparticles (AgNPs) (0.4%) on dentin), and group 4 (apply GIC with silver nanoparticles (AgNPs) (0.6%) on dentin). For the preparation of the control group, the ratio of powder and liquid was taken as per the manufacturers’ instructions, and they were mixed on a glossy paper pad. Subsequently, all the samples were prepared for FTIR.

### Analysis of the Mechanical Interaction

Fourier-transform infrared spectroscopy (FTIR) (Figure 1) provides the vibrational modes of the molecules, evaluated by the optical absorption bands of the sample, which are considered the fingerprints of specific molecules, enabling precise information about chemical changes in the material, the latter being assessed based on the possible changes in absorption bands and/or the appearance of new bands (Larkin, 2011). Teeth with 0.2%, 0,4%, and 0.6% w/w of silver nanoparticles and GIC, the control tooth, and plain silver were subjected to FTIR analysis using an ATR–FTIR spectrophotometer (Thermo Fisher Scientific, Waltham, MA, United States) with zinc selenide (ZnSe) ATR crystals (attenuated total reflection) and OPUS v7.5 software. All spectra were recorded in the range of 500–3,500 cm^−1^ in the transmission mode with an ATR module. The FTIR vibration range mode wavenumber was from 500 to 3500 cm^−1^. The FTIR analysis of GIC showed a similar interaction with the dentin compared to the GIC, with 0.2 and 0.4% AgNPs. These vibrational groups were part of the cross-linking reaction and aging time. In addition, the FTIR spectra showed the vibration of Ag in molecular water associated at the range of 3300 cm^−1^. The vibration band then shifted to 880 cm^−1^. This band was related to the bonding structure present in the GIC sample with 0.6%. The other band existing at ∼1,550 cm^-1^ referred to the formation of the asymmetric COOH band from the PAA.

### Statistical Analysis

Only descriptive analysis was carried out, and statistical analysis was not performed in this study due to the qualitative characteristics of the data resulting from FTIR (Yamakami et al., 2018).

## Results

**FIGURE 2:**
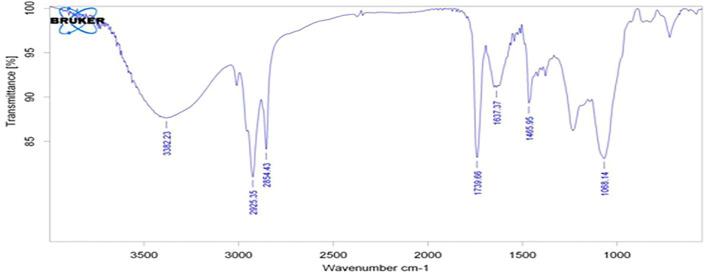
Bioactive evaluation of silver nanoparticles.

**FIGURE 3:**
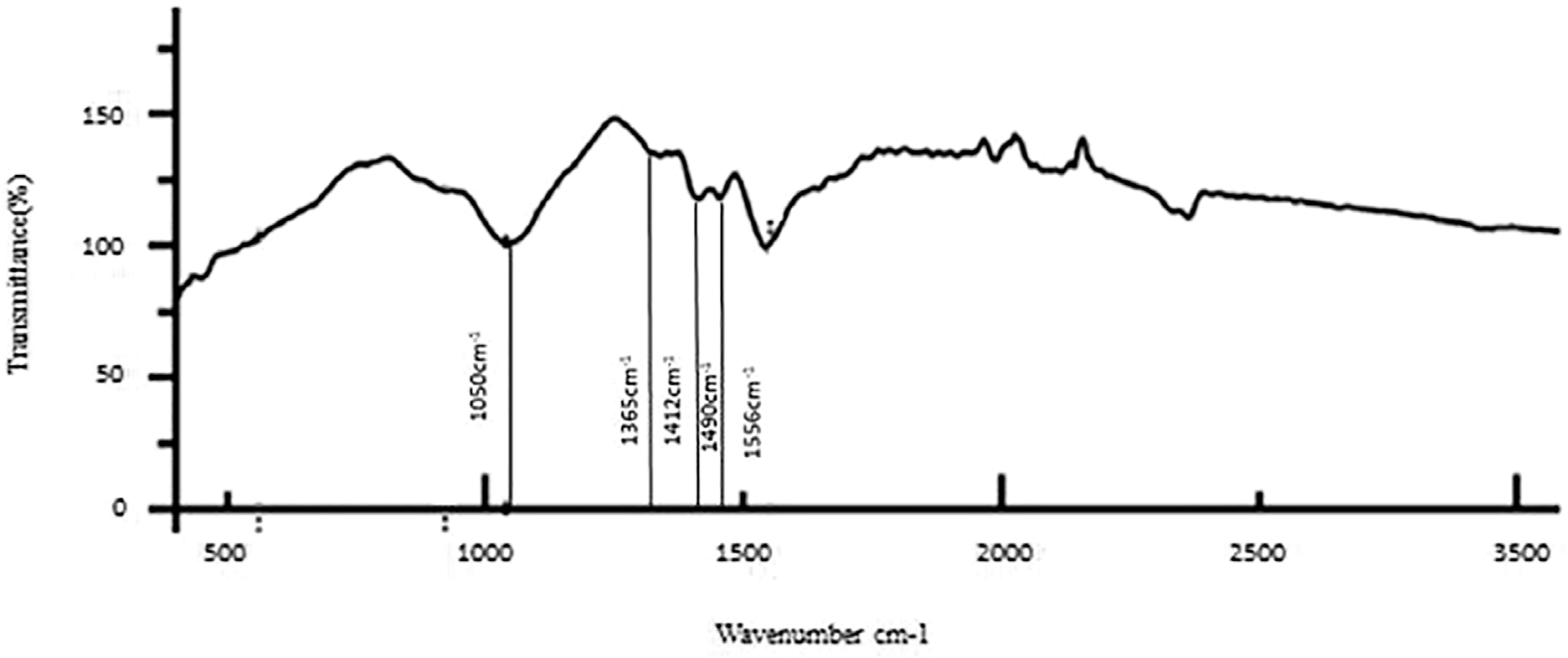
Bioactive evaluation of glass ionomer cement (GIC) with dentin.

**FIGURE 4:**
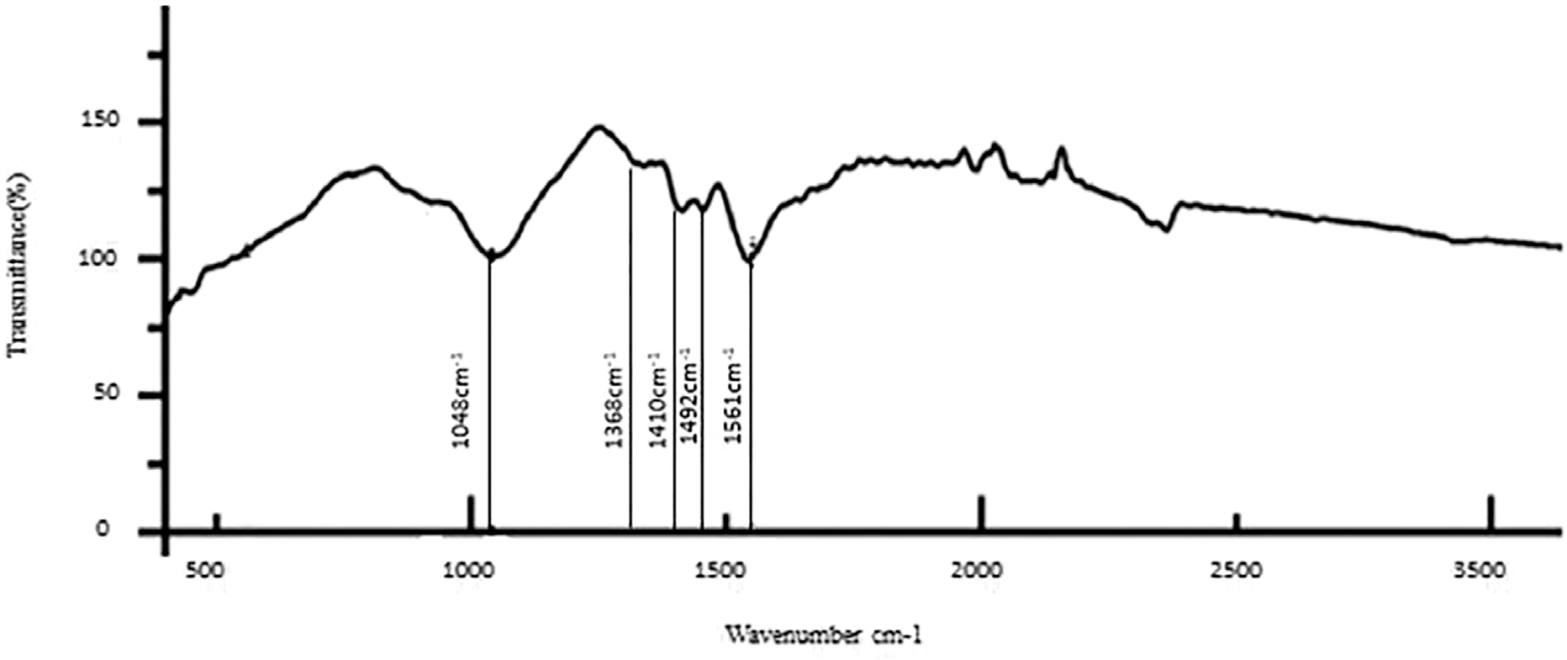
Bioactive evaluation of glass ionomer cement (GIC) and silver nanoparticles (AgNPs) 0.2% sample with dentin.

The results of the bioactive evaluation of silver nanoparticle cement, carried out by Fourier-transform infrared spectroscopy, are shown in Figure 2. The GIC had various peaks, of which v1, v2, v3, v4, and v5 with 1068 cm^−1^, 1365 cm^−1^, 1456 cm^−1^, 1637 cm^−1^, and 1740 cm^−1^, respectively, were significant. The results of the bioactive evaluation of glass ionomer cement, carried out by Fourier-transform infrared spectroscopy, are shown in Figure 3. The GIC had various peaks, of which v1, v2, v3, v4, and v5 with 1050cm^−1^, 1,365 cm^−1^, 1,412 cm^−1^, 1490 cm^−1^, and 1556 cm^−1^, respectively, were significant. The GIC with silver nanoparticles (AgNPs) 0.2% had various peaks, of which v1, v2, v3, v4, and v5 with 1,048 cm^−1^, 1,368 cm^−1^, 1,410 cm^−1^, 1492 cm^−1^, and 1561 cm^−1^, respectively, were significant. The results of the bioactive evaluation of glass ionomer cement and silver nanoparticles (AgNPs) 0.2% with dentin, carried out by Fourier-transform infrared spectroscopy, are shown in Figure 4.

**FIGURE 5:**
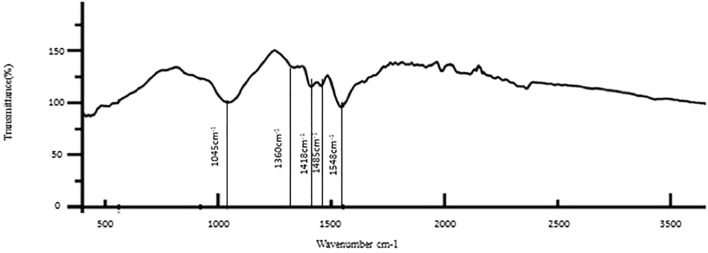
Bioactive evaluation of glass ionomer cement (GIC) and silver nanoparticles (AgNPs) 0.4% sample with dentin.

**FIGURE 6:**
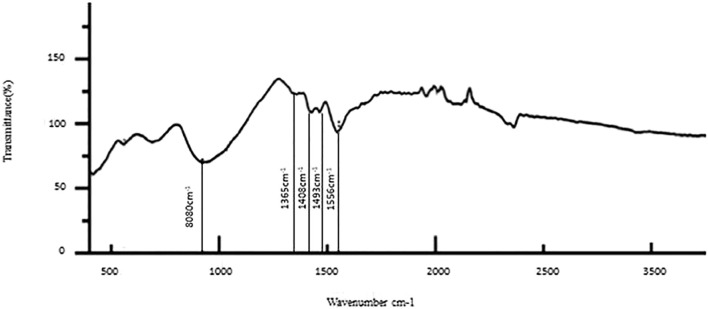
Bioactive evaluation of glass ionomer cement (GIC) and silver nanoparticles (AgNPs) 0.6% sample with dentin.

The results of the bioactive evaluation of glass ionomer cement with silver nanoparticles (AgNPs) 0.4% with dentin, carried out by Fourier-transform infrared spectroscopy, are shown in Figure 5. The GIC with silver nanoparticles (AgNPs) 0.4% had various peaks, of which v1, v2, v3, v4, v5 with 1,045 cm^−1^, 1,360 cm^−1^, 1,418 cm^−1^, 1,485 cm^−1^, and 1,548 cm^−1^, respectively, were significant. The GIC with silver nanoparticles (AgNPs) 0.6% had various peaks, of which v1, v2, v3, v4, and v5 with 8,080 cm^−1^, 1,365 cm^−1^, 1,408 cm^−1^, 1,493 cm^−1^, and 1,565 cm^−1^, respectively, were significant. The results of the bioactive evaluation of glass ionomer cement with silver nanoparticles (AgNPs) 0.6% with dentin, carried out by Fourier-transform infrared spectroscopy, are shown in Figure 6. The results of the bioactive evaluation of dentin performed by Fourier-transform infrared spectroscopy are shown in Table 1. The dentin had various peaks, of which v1, v2, v3, and v4, with 1040 cm^−1^, 1242 cm^−1^, 1546 cm^−1^, and 1655 cm^−1^, respectively, were significant.

**TABLE 1:** Wave numbers of dentin by Fourier-transform infrared spectroscopy.

Tissue	Amide I	Amide II	Amide III	Phosphate
Dentin	1,655	1,546	1,242	1,040

## Discussion

The present study has been carried out to investigate the effect of the bonding interaction of AgNP incorporation into GIC onto the dentin surface. Three concentrations (0.2, 0.4, and 0.6%) were added, with the control being the GIC. This study showed a greater variation of the transmission bands after the increased addition of silver nanoparticles, indicating a change in bond interaction with the dentin. Pure GIC has the disadvantage of less wear resistance, and it cannot withstand the masticatory forces. The common reason for the low resistance of GIC to fracture is the presence of voids in the cement matrix, which are formed by the inclusion of air during cement mixing. These voids may act as stress raisers and concentrators and eventually weaken the mechanical properties of the set cement (Kent, 1973; Elsaka et al., 2011; Liu et al., 2014). Many practitioners use this restoration due to the major advantage of its fluoride-releasing property (Xu and Burgess, 2003). The manufacturers also release many combinations to improve the mechanical properties without losing the fluoride release.

Recent studies suggest that the voids tend to be filled with nanoparticles incorporated into GIC (Elsaka et al., 2011; Gjorgievska et al., 2015). In this process of experimentation, a limited number of studies were carried out incorporating SNP with GIC (Paiva et al., 2018; Jowkar et al., 2019). Jowkar and co-workers (2019) used the addition of 0.1 and 0.2% of AgNPs to GIC in their study and concluded that the higher concentration of 0.2% showed a significant improvement in mechanical properties (surface hardness, flexural strength, compressive strength, and micro-shear bond strength to dentin). Paiva and co-workers (2018) concluded that a higher concentration of silver (0.5% by mass) in the matrix of nano-Ag-GIC allowed viable net setting time and increased the compressive strength of the experimental cement by 32%. The addition of AgNPs increased the mechanical properties of the GIC and improved the antibacterial property in arresting the caries (Padovani et al., 2015; Raggio et al., 2016; Paiva et al., 2018; Nuvvula and Mallineni, 2019).

The small sizes of the silver nanoparticles incorporated into GIC and the improved packing of particles within the matrix of the set cement may explain the improvement of the flexural and compressive strengths of the AgNP-containing GIC. Incorporating AgNPs into GIC may also result in a broader particle size distribution range. Therefore, these small silver nanoparticles can occupy the empty spaces between the larger glass particles and provide an additional bonding site for the polyacrylic polymer (Moshaverinia et al., 2008; Moshaverinia et al., 2010). Considering all these factors, we completed novel research on how various concentrations of AgNPs would change the interaction of GIC and AgNPs with the dentin. Several studies (Paiva et al., 2018; Jowkar et al., 2019) have shown that any concentration less than 0.5% AgNPs with GIC improved the mechanical properties. Hence, our study added more than 0.5% AgNPs, that is, 0.6% AgNPs as one of the groups along with 0.2 and 0.4%.

The present study aimed to examine the quality of the bonding interaction without changing the ideal bond quality achieved with GIC. However, the study focused on the exchanges that occurred when the cement was brought into contact with dentin. It is essential to differentiate between short-term and long-term interactions. Short-term interactions occur when the freshly prepared glass ionomer cement is brought into contact with dentin. They correspond to the rapid inter-diffusions between the dentin elements and the glass ionomer cement when the cement is not entirely set. These inter-diffusions enable the GIC to adhere to dentin. This ceases once the cement has been developed completely. Long-term interactions correspond to the slow diffusion of some elements of the glass ionomer cement through dentin. They can be caused by water in the buccal environment (Sennou et al., 1999).

This quality of binding of any restorative material to dentin is achieved through various methods such as FTIR (Paiva et al., 2018; Yamakami et al., 2018; Jowkar et al., 2019), Raman spectroscopy (Larkin, 2011; Yamakami et al., 2018), infrared spectroscopy (Larkin, 2011), and X-ray photoelectron spectroscopy (Sennou et al., 1999). Devaraj and co-workers (2013) reported that the FTIR spectra of silver nanoparticles exhibited prominent peaks at 2,927 cm^−1^, 1,631 cm^−1^, and 1,383 cm^−1^. Similar peaks were evident in the present study with little variation, showing various peaks, such as v1, v2, v3, v4, and v5 with 1,068 cm^−1^, 1365 cm^−1^, 1456 cm^−1^, 1637 cm^−1^, and 1740 cm^−1^, respectively. FTIR of the dentin surface showed several amide peaks (amide I, amide II, and amide III) in the range between 1,250 and 1,650 cm^−1^, and the phosphate intensity ranged slightly over 1,000 cm^−1^ (Table 1). These results are similar to Spencer et al. (2005); Cao et al. (2014). Lin and co-workers (2001) reported two different absorption bands at 2,200 cm^−1^ and 2015 cm^−1^ in the spectrum. Lopes and co-workers (2018) suggested a reaction in the organic matrix or between the organic matrix and minerals, resulting in a different peak.

The phosphate bonds were more peculiar with four vibrational modes: v1, v2, v3, and v4. All these modes were infrared radiography active and observed in dentin. In the present study, a single intense v3 band was observed at about 1046 cm^-1^. The v3 band overlapped with the v1 band, the first one of greater intensity (Nelson and Featherstone, 1982). The phosphate v1 band was present at 960 cm^−1^. The phosphate v4 band was observed at in 660 cm^1^ and 520 cm^1^ and was a sharp, well-defined band (Rehman and Bonfield, 1997). Last, a soft phosphate v2 band was observed in the region of 470 cm^−1^ (Bachmann et al., 2003). In the present study, AgNPs were added at 0.2, 0.4, and 0.6% concentration to GIC and provided evidence for the context of bond interaction with the dentin. There was a clear shift evident in the phosphate peak for control, 0.2%, and 0.4%, which was around 1050 cm^−1^, while for 0.6%, there was a clear shift from 1050 cm^−1^ to 880 cm^−1^, which was evident in the present study. Various bond peaks were seen for calcium, carbonate, phosphate, and amide. In our study, only the amide and phosphate groups significantly generated peaks. The amide peaks were similar to the control, 0.2%, 0.4%, and 0.6%, ranging from 1250 to 1650 cm^−1^. This shows that there was a change in the interaction of bonding. We found a change in bond quality when AgNPs increased to 0.6% in the present study.

### Limitations

The statistical analysis was not carried out in the present study, based on the study and descriptive analysis carried out by Yamakami et al. (2018), and this is also considered one of the potential limitations. It was an *in vitro* study, and we cannot assess what would happen in a clinical setting. Second, we used GIC GC Fuji II in the study, and variations may occur using other types of GIC. FTIR does not offer the high spatial resolution capabilities of different techniques such as micro-Raman spectroscopy (approx. 1 μm). However, FTIR has the advantage that IR spectra, with an acceptable signal/noise ratio, can be collected from areas measuring several hundred square micrometers in a matter of minutes.

### Recommendations

We warrant further research to examine the addition of other substances to GIC and their effect on the bond strength of this material. *In vitro* studies already have good evidence, but we suggest *in vivo* studies to improve the quality of the restorations in a clinical condition.

## Conclusion

The descriptive analysis in the present study showed that any concentration beyond 0.4% of AgNPs altered the bond quality with dentin interaction. In conclusion, adding AgNPs to a minimum improves the mechanical properties and maintains the same bond quality as GIC.

## Data Availability

The raw data supporting the conclusion of this article will be made available by the authors, without undue reservation.

## Abbreviations

Ag, silver; AgNO3, silver nitrate; AgNPs, silver nanoparticles; Al2o3, aluminum oxide; ATR, attenuated total reflection; Bis-GMA, bisphenol A-glycidyl methacrylate; CaF2, calcium fluoride; CaO, calcium oxide; COPD, chronic obstructive pulmonary disease; FTIR, Fourier-transform infrared spectroscopy; GIC, glass ionomer cement; HA, hydroxyapatite; HEMA, hydroxyethyl methacrylate; ISO, International Organization of Standardization; NaBo4, sodium perborate; NPs, nanoparticles; P2o5, phosphorus pentoxide; PAA, polyacrylic acid; *S. aureus*, *Staphylococcus aureus*; Sio2, silicon dioxide; SioH, silanol; ZnSe, zinc selenide.
